# Switching to Integrase Inhibitors and Cardiovascular Disease Risk in Treatment-Experienced People With HIV: A Sequential Target Trial Emulation in the Swiss HIV Cohort Study

**DOI:** 10.1093/ofid/ofag479

**Published:** 2026-08-07

**Authors:** Tristan T Lee, Matthias Cavassini, Annalisa Marinosci, David Haerry, Marcel Stöckle, Patrick Schmid, Luigia Elzi, Christoph A Fux, Philip E Tarr, Lukas Baumann, Huldrych F Günthard, Gilles Wandeler, Niklaus D Labhardt, Bernard Surial, Frédérique Chammartin, the Swiss HIV Cohort Study, I A Abela, K Aebi-Popp, A Anagnostopoulos, E Bernasconi, D L Braun, H C Bucher, A Calmy, M Cavassini, A Ciuffi, G Dollenmaier, M Egger, L Elzi, J S Fehr, J Fellay, S Frigerio Malossa, C A Fux, H F Günthard, A Hachfeld, D H U Haerry, B Hasse, H H Hirsch, M Hoffmann, I Hösli, M Huber, D Jackson-Perry, C R Kahlert, D Kaufmann, O Keiser, T Klimkait, R D Kouyos, H Kovari, K Kusejko, N D Labhardt, K Leuzinger, B Martinez de Tejada, C Marzolini, K J Metzner, N Müller, J Nemeth, D Nicca, J Notter, P Paioni, G Pantaleo, M Perreau, A Rauch, L P Salazar-Vizcaya, P Schmid, O Segeral, R F Speck, M Stöckle, B Surial, P E Tarr, A Trkola, G Wandeler, M Weisser, S Yerly

**Affiliations:** Division of Clinical Epidemiology, Department of Clinical Research, University Hospital Basel, University of Basel, Basel, Switzerland; Division of Infectious Diseases, University Hospital of Lausanne, University of Lausanne, Lausanne, Switzerland; Division of Infectious Diseases, Geneva University Hospital, University of Geneva, Geneva, Switzerland; Chair Positive Council, Zurich, Switzerland; Division of Infectious Diseases and Hospital Epidemiology, University Hospital Basel, University of Basel, Basel, Switzerland; Division of Infectious Diseases, Infection Prevention and Travel Medicine, HOCH Health Ostschweiz, Cantonal Hospital St Gallen, University Teaching and Research Hospital, St Gallen, Switzerland; Division of Infectious Diseases, Ente Ospedaliero Cantonale, Regional Hospital of Bellinzona and University of Basel, Basel, Switzerland; Division of Infectious Diseases, Cantonal Hospital of Aarau, Aarau, Switzerland; University Center for Internal Medicine and Division of Infectious Diseases and Hospital Epidemiology, Kantonsspital Baselland, University of Basel, Basel, Switzerland; Department of Infectious Diseases, Inselspital, Bern University Hospital, University of Bern, Bern, Switzerland; Department of Infectious Diseases and Hospital Epidemiology, University Hospital Zurich, University of Zurich, Zurich, Switzerland; Institute of Medical Virology, University of Zurich, Zurich, Switzerland; Department of Infectious Diseases, Inselspital, Bern University Hospital, University of Bern, Bern, Switzerland; Division of Clinical Epidemiology, Department of Clinical Research, University Hospital Basel, University of Basel, Basel, Switzerland; Department of Infectious Diseases, Inselspital, Bern University Hospital, University of Bern, Bern, Switzerland; Division of Clinical Epidemiology, Department of Clinical Research, University Hospital Basel, University of Basel, Basel, Switzerland

**Keywords:** antiretroviral therapy, integrase strand transfer inhibitor, myocardial infarction, stroke, treatment switch

## Abstract

**Background:**

Integrase strand transfer inhibitors (INSTIs) are highly effective components of antiretroviral therapy for HIV. Recent studies suggest increased cardiovascular disease (CVD) risk after INSTI initiation, but findings remain inconsistent. The Swiss HIV Cohort Study is nationally representative, collects high-quality data, and provides an ideal setting for independent evaluation using causal methods, avoiding participant overlap in multicountry HIV-cohort consortiums.

**Methods:**

We emulated sequential target trials from November 2011 to September 2025 to estimate the effect of switching from non-INSTI to INSTI-based treatment on 6-year CVD risk among treatment-experienced people with HIV. Eligible participants were ≥18 years old, INSTI-naive, virally suppressed, and without CVD history. For each trial, participants were assigned to remain on non-INSTI or switch to INSTI. Intention-to-treat (ITT) and per-protocol (PP) effects were estimated using pooled logistic regression with baseline adjustment (ITT) and inverse probability weighting (PP).

**Results:**

Among 8815 individuals contributing 539 017 person-trials, 5750 switched to INSTI, and 507 CVD events occurred. In ITT analyses, the adjusted risk ratio for switching versus remaining on non-INSTI was 1.34 (95% confidence interval 1.03–1.69) after 1 year, and 1.03 (0.88–1.16) after 6 years. Absolute risk differences were small, ranging from 0.20 percentage points (0.02–0.37) after 1 year to 0.13 (-0.49 to 0.58) after 6 years. Per-protocol estimates were similar.

**Conclusions:**

Switching to INSTI-based treatment did not substantially increase long-term CVD risk in treatment-experienced individuals with well-controlled HIV. The early increase in absolute risk was small and was not expected to be clinically meaningful. This further contextualizes results from multicountry analyses.

The introduction of integrase strand transfer inhibitors (INSTIs) represents a major advance in contemporary antiretroviral therapy (ART) for people with HIV (PWH). Compared with other drug classes, INSTIs are highly effective, and second-generation INSTIs, such as dolutegravir and bictegravir, have a high barrier to resistance [1–3]. Since the approval of raltegravir, the first INSTI, in 2007, their use has expanded rapidly, and INSTIs now form the backbone of most combination treatments worldwide [4].

Recent evidence has suggested that INSTIs may be associated with an increased risk of cardiovascular disease (CVD), which is of particular concern as the population with HIV ages [5]. A 2022 analysis of the International Cohort Consortium of Infectious Diseases (RESPOND) reported an increased risk of CVD within the first 24 months of INSTI exposure, with differences in risks becoming negligible thereafter [6]. The biological mechanism underlying this early risk elevation remains unclear. Interpretation of the RESPOND findings is further limited by the noncausal study design and by potential biases related to stratified participant selection in this multinational cohort.

In response to these findings, several causal analyses, including target trial emulations using observational data, have been conducted across diverse cohorts and settings. Among studies in people newly initiating ART, findings consistently point toward no increased risk of CVD [7–9]. Evidence among treatment-experienced populations is less consistent. For example, Rein et al [8] found no difference in CVD risk among individuals with well-controlled HIV 4 years after switching to INSTI-based ART compared with those who remained on non-INSTI–based ART, although cumulative incidence curves suggested a modestly higher incidence in the early period following the switch.

The Swiss HIV Cohort Study (SHCS) contributed a substantial proportion of treatment-experienced individuals for the multinational collaboration analyses (RESPOND and ART-CC). While large pooled analyses provide results that may be more generalizable over different settings and health systems, analyses from a single setting with high-quality data and comparatively consolidated clinical practices may help to understand specific trends or biological mechanisms. The SHCS provides a robust and standardized assessment of CVD outcomes within a large, nationally representative sample of PWH receiving ART in Switzerland and is therefore well positioned to reproduce these analyses and contextualize their findings. In this study, we assessed the effect of switching to an INSTI-based ART treatment versus remaining on non-INSTI–based ART on the 6-year risk of CVD among well-controlled PWH in the SHCS.

## METHODS

### Study Design and Data

We conducted an observational study emulating a sequence of target trials to estimate the causal intention-to-treat (ITT) and per-protocol (PP) effects of switching to INSTI-based ART compared with remaining on non-INSTI–based ART on CVD events in the SHCS. Repeated target trials were emulated at the start of each calendar month between November 2011 and September 2025. November 2011 is when the European AIDS Clinical Society guidelines first recommended INSTIs as part of first-line ART [10]. Each monthly time point was treated as a baseline of a hypothetical trial, appropriately aligning eligibility, treatment assignment, and start of follow-up and reducing time-related biases [11]. Individuals could be included in multiple trials if they met the eligibility criteria and, therefore, could contribute CVD events more than once. This approach is particularly relevant for treatment-experienced PWH, who may become eligible for switching ART at multiple follow-up visits.

We applied the target trial framework, which involves (1) specifying the protocol for the randomized controlled trial we would hypothetically conduct and (2) emulating this trial using observational data [12]. In brief, we conceptualized a target trial enrolling virally suppressed (≤50 copies/mL) PWH above 18 years old on a non-INSTI–based ART (≥3 antiretrovirals from ≥2 classes or protease inhibitor [PI]-based dual therapy) and without history of CVD. Participants would be randomly allocated to 1 of 2 treatment strategies: (1) INSTI-based ART, defined as an ART combination including at least 1 INSTI and (2) non-INSTI–based ART, defined as an ART combination not including an INSTI. The outcome of interest was the risk of CVD, defined as the first occurrence of myocardial infarction, stroke, or an invasive cardiovascular procedure, over 6 years of follow-up. Detailed eligibility criteria, treatment strategies, outcome, and follow-up are provided in the Appendix, with the full target trial protocol summarized in Supplementary Table 1.

To emulate the trial, we used observational data from the SHCS, an ongoing prospective cohort study that includes ∼72% of PWH receiving ART in Switzerland. The SHCS routinely collects sociodemographic, behavioral, clinical, and laboratory data, as well as complete ART and comedication histories, at enrollment and regular follow-ups, using standardized protocols [5]. Currently, follow-ups occur at 6-month (90% of participants) or 12-month (10% of participants) intervals. Cardiovascular events are centrally validated using standardized algorithms and adjudicated blinded to patients’ ART history [13]. The cohort study was approved by the ethics committees of all participating centers, and all patients provided written informed consent. A patient representative contributed to both the study and manuscript development and is listed as a co-author. This study adheres to the TARGET guidelines for reporting of target trial emulation studies using observational data to estimate causal effects [14].

### Statistical Analysis

Sequential trials were emulated and analyzed using pooled logistic regressions. The ITT causal contrast of interest estimated the risk of CVD according to treatment assignment at the start of each trial, while the PP effect compared outcomes under sustained adherence to the assigned treatment strategy, artificially censoring individuals when they deviated from it.

The ITT analysis was performed on a sequence of 167 trials initiated at the start of each month of the study period. A person-month dataset was generated by carrying forward time-varying characteristics between cohort visits. Missing data were treated as distinct categories. At each trial initiation, individuals’ baseline characteristics were set to the observed values at that specific start month, eligibility was assessed, and the treatment arm was assigned to be compatible with the individual's observed data. For each sequential trial, participants meeting the target trial eligibility criteria were followed until a CVD event or censoring due to death, loss to follow-up, administrative database closure or until the end of the trial, whichever came first. We assumed that switching to an INSTI-based ART was random within levels of baseline covariates. Thus, our model was adjusted for baseline covariates to control for confounding. Our full pooled logistic regression model included treatment assignment, an interaction between treatment and time, where time was modeled linearly and quadratically and the following confounders: age, sex, education (high level, basic, no professional education, and unknown), race (White, Black, other, and unknown), HIV transmission risk (heterosexual contacts, people who inject drugs, men who have sex with men, and other), family history of CVD (yes/no), years on ART, smoking status (yes/no), body mass index (BMI; underweight [<18.5 kg/m^2^], normal [18.5–24.9 kg/m^2^], overweight [25–29.9 kg/m^2^], obese [≥30 kg/m^2^], and unknown), CD4 cell count (≥500 cells/µL, 350–499 cells/µL, 200–349 cells/µL, <200 cells/µL, and missing), CD4 nadir (≥500 cells/µL, 350–499 cells/µL, 200–349 cells/µL, <200 cells/µL, and missing), total cholesterol (<4.1 mmol/L, 4.1–5.19 mmol/L, 5.2–6.19 mmol/L, 6.2–7.2 mmol/L, >7.2 mmol/L, and missing), estimated glomerular filtration rate (≥90 mL/min, 60–89 mL/min, <60 mL/min, and missing), hypertension, diabetes, current tenofovir alafenamide (TAF) use, current abacavir (ABC) use, current antiplatelet drug use, current lipid-lowering drug use, and baseline calendar month (linear and quadratic). Hypertension was defined as having a most recent blood pressure measurement >140/90 mm Hg and/or currently being on an antihypertensive medication, while diabetes was defined as having a diabetes diagnosis or starting an antidiabetic drug. Estimated glomerular filtration rate was calculated using the chronic kidney disease epidemiology collaboration (CKD-EPI) equation [15].

In the PP analysis, participants were artificially censored upon deviation from their assigned treatment strategy, defined as switching to the alternative treatment arm, or stopping ART for >2 months. To adjust for confounding and selection bias from censoring, we derived time-varying nonstabilized inverse probability weights to address (1) artificial censoring due to treatment nonadherence and (2) other forms of censoring listed above. For nonadherence to treatment, we developed a pooled logistic regression for treatment adjusting for the same confounders as in the ITT outcome model analysis and time as calendar month. For other forms of censoring, we developed a pooled logistic regression for being censored, adjusting for similar confounders, the treatment, and time as calendar month. Both models were fitted using our original longitudinal dataset, and probabilities were predicted for each time point and inverted. These values were subsequently assigned to each person-trial according to individual identifier, trial baseline, and follow-up time. Weights were derived by taking the cumulative inverted predicted probability values, giving a unique weight for each person-trial and follow-up month. Final weights were truncated at the 99th percentile to mitigate disproportionate influence on our estimates. Similarly to the ITT analysis, the outcome model included an interaction between treatment and time, where time was modeled linearly and quadratically. Thus, confounding in the PP analysis was not controlled through covariate adjustment, but inverse probability weights created a pseudo-population in which adherence was independent of measured confounders.

Intention-to-treat and PP pooled logistic models were used to predict CVD hazard under each treatment strategy. Cumulative risk was averaged across all sequential trials to estimate the population-average treatment effects, expressed as adjusted risk ratios (aRRs) and adjusted risk differences (aRDs), together with 95% confidence intervals (CIs) derived from nonparametric bootstrapping with 200 samples. Analysis was completed using R version 4.4.3.

### Subgroup and Sensitivity Analysis

Exploratory subgroup analyses were conducted to assess whether the effect of switching to INSTI-based ART on CVD risk varied across patient groups with different baseline risk or treatment patterns. We considered subgroups for sex, age group (<65 vs ≥65 years old), and whether or not ABC, which has previously been associated with CVD risk, was part of the trial-specific baseline treatment [16, 17]. For each subgroup, we fit the same models as in the ITT analysis and report risk differences and ratios between those on INSTI-based ART compared with non-INSTI–based ART, with CIs calculated using robust standard errors. To formally assess for effect modification, we included an interaction term between treatment and subgroup in the pooled ITT model. We considered *P*-values associated with interaction term <0.1 suggestive for effect modification. Because raltegravir was initially used as a rescue medication, we conducted a sensitivity analysis excluding individuals who initiated raltegravir and censoring at the time of raltegravir switch.

## RESULTS

**Figure 1.**
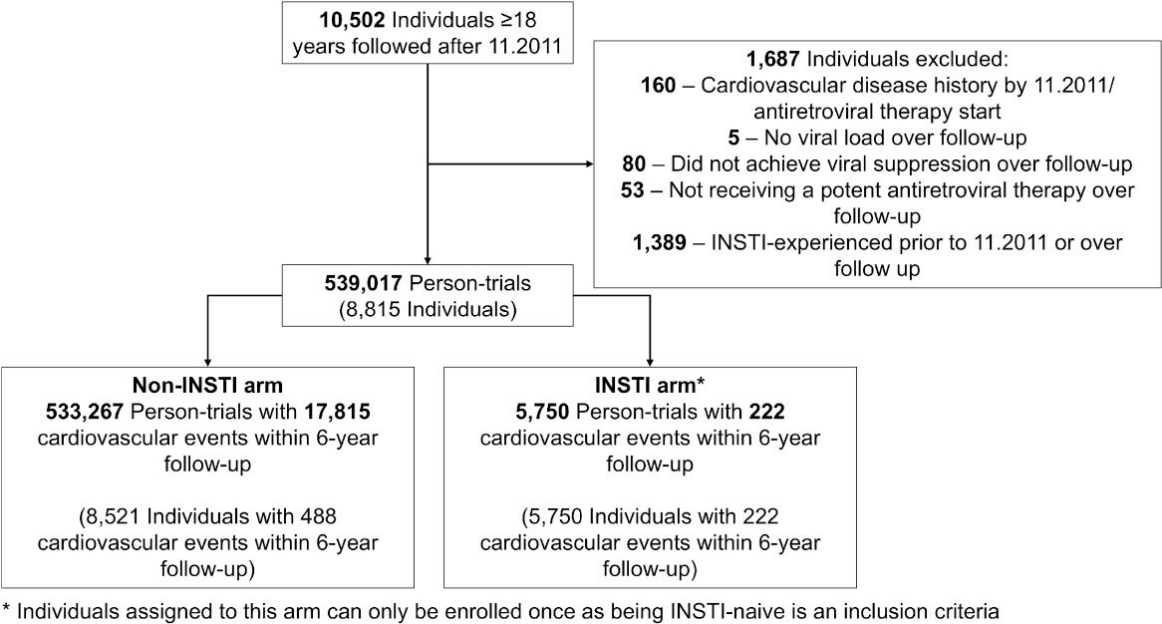
Selection of antiretroviral-experienced people with HIV for sequential target trial emulation. The figure reports number of individuals and number of person-trials participated in.

A total of 10 502 individuals were followed in the SHCS from November 2011 onwards. Of these, 1687 were excluded because of having a history of CVD by the start of the first trial (n = 160), having no viral load (n = 5), never achieving viral suppression (n = 80), not being on ART (n = 52), and being INSTI experienced (n = 1389). Overall, 8815 individuals were included for analysis. These individuals contributed to a total of 539 017 person-trials, of which 533 267 were assigned to the non-INSTI strategy, and 5750 were assigned an INSTI-based strategy (Figure 1). The median number of trials per participant was 51 (interquartile range [IQR]: 28–86). Overall, 30.3% of participants were female and 16.0% were of African ancestry (Table 1). The main modes of HIV acquisition were through men having sex with men (44.0%) and heterosexual contact (40.6%). At trial baseline, the median age was 48 years (IQR: 41–55), the median year of ART initiation was 2005 (IQR: 1999–2010), and the median time on ART was 11 years (IQR: 6–16). The median CD4 cell count was 631 cells/µL (IQR: 473–826). Baseline characteristics including BMI, CD4 cell count, cholesterol, hypertension, diabetes, smoking, and taking a lipid-lowering therapy were similar between individuals who switched to INSTI-based ART and those who remained on non-INSTI–based ART. Use of TAF (31.4% vs 15.9%) and ABC (33.1% vs 20.2%) was more frequent in the INSTI-based ART arm compared with the non-INSTI–based ART arm. Of the 5750 participants who switched to INSTI, 3249 received dolutegravir (56.8%), 1040 elvitegravir (17.9%), 711 bictegravir (12.3%), 679 raltegravir (11.8%), and 71 cabotegravir (1.2%). Baseline ART classes and the most common ART regimens are shown in Supplementary Tables 2 and 3. We observed 963 individuals who subsequently switched from an INSTI back to a non-INSTI–based ART over follow-up.

**Table 1.** Baseline Characteristics Across All Sequential Target Trials, by Treatment Strategy and Overall, With Standardized Mean Differences

	Overall (N = 539 017)	Remaining on Non-INSTI–Based ART (N = 533 267)	Switching to INSTI-Based ART (N = 5 750)	Standardized Mean Difference
Male sex, n (%)	375 961 (69.7)	371 930 (69.7)	4031 (70.1)	0.008
Age in years, median (IQR)	48 (41–55)	48 (41–55)	50 (42.2–56)	0.089
Years on ART, median (IQR)	11.0 (6.0–16.0)	11.0 (6.0–16.0)	12.0 (7.0–18.0)	0.142
Year ART started, median (IQR)	2005 (1999–2010)	2005 (1999–2010)	2005 (1998–2010)	0.001
Race, n (%)				
White	403 240 (74.8)	398 899 (74.8)	4341 (75.5)	0.024
African ancestry	86 218 (16.0)	85 340 (16.0)	878 (15.3)	…
Other	48 749 (9.0)	48 224 (9.0)	525 (9.1)	…
Unknown	810 (0.2)	804 (0.2)	6 (0.1)	…
Highest education achieved, n (%)				
High-level education	174 931 (32.5)	173 109 (32.5)	1822 (31.7)	0.040
Basic education	312 507 (58.0)	309 105 (58.0)	3402 (59.2)	…
No professional education	39 269 (7.3)	38 892 (7.3)	377 (6.6)	…
Unknown	12 310 (2.3)	12 161 (2.3)	149 (2.6)	…
HIV transmission group, n (%)				
Heterosexual	218 701 (40.6)	216 505 (40.6)	2196 (38.2)	0.051
Men having sex with men	237 014 (44.0)	234 412 (44.0)	2602 (45.3)	…
Person who injects drugs	57 863 (10.7)	57 201 (10.7)	662 (11.5)	…
Other	25 439 (4.7)	25 149 (4.7)	290 (5.0)	…
Family history of CVD, n (%)	53 269 (9.9)	52 646 (9.9)	623 (10.8)	0.039
Body mass index, n (%)				
Underweight (<18.5 kg/m^2^)	18 842 (3.5)	18 629 (3.5)	213 (3.7)	0.020
Normal (18.5–24.9 kg/m^2^)	276 105 (51.2)	273 130 (51.2)	2975 (51.7)	…
Overweight (25–29.9 kg/m^2^)	161 586 (30.0)	159 894 (30.0)	1692 (29.4)	…
Obese (≥30 kg/m^2^)	62 272 (11.6)	61 605 (11.6)	667 (11.6)	…
Unknown	20 212 (3.7)	20 009 (3.8)	203 (3.5)	…
Currently smoking, n (%)				
Yes	186 163 (34.5)	184 127 (34.5)	2036 (35.4)	0.021
No	332 951 (61.8)	329 436 (61.8)	3515 (61.1)	…
Missing	19 903 (3.7)	19 704 (3.7)	199 (3.5)	…
CD4 cell count (cells/μL), median (IQR)]	631.0 (473.0, 826.0)	631.0 (473.0, 826.0)	643.0 (474.0, 835.0)	0.015
CD4 cell count, n (%)				
≥500 cells/μL	381 566 (70.8)	377 487 (70.8)	4079 (70.9)	0.073
350–499 cells/μL	101 148 (18.8)	100 132 (18.8)	1016 (17.7)	…
200–349 cells/μL	44 599 (8.3)	44 133 (8.3)	466 (8.1)	…
<200 cells/μL	10 264 (1.9)	10 098 (1.9)	166 (2.9)	…
Missing	1440 (0.3)	1417 (0.3)	23 (0.4)	…
CD4 cell count nadir, n (%)				
≥500 cells/μL	36 507 (6.8)	36 077 (6.8)	430 (7.5)	0.048
350–499 cells/μL	66 908 (12.4)	66 222 (12.4)	686 (11.9)	…
200–349 cells/μL	189 221 (35.1)	187 294 (35.1)	1927 (33.5)	…
<200 cells/μL	245 362 (45.5)	242 670 (45.5)	2692 (46.8)	…
Missing	1019 (0.2)	1004 (0.2)	15 (0.3)	…
Arterial hypertension, n (%)				
Yes	148 710 (27.6)	147 013 (27.6)	1697 (29.5)	0.043
Diabetes, n (%)				
Yes	26 927 (5.0)	26 616 (5.0)	311 (5.4)	0.019
Total cholesterol, n (%)				
<4.1 mmol/L	83 218 (15.4)	82 350 (15.4)	868 (15.1)	0.042
4.1–5.19 mmol/L	203 677 (37.8)	201 574 (37.8)	2103 (36.6)	…
5.2–6.19 mmol/L	155 495 (28.8)	153 787 (28.8)	1708 (29.7)	…
6.2–7.2 mmol/L	62 655 (11.6)	61 973 (11.6)	682 (11.9)	…
>7.2 mmol/L	16 281 (3.0)	16 075 (3.0)	206 (3.6)	…
Missing	17 691 (3.3)	17 508 (3.3)	183 (3.2)	…
HDL cholesterol, n (%)				
>1.6 mmol/L	130 136 (24.1)	128 805 (24.2)	1331 (23.1)	0.057
1.3–1.6 mmol/L	148 373 (27.5)	146 864 (27.5)	1509 (26.2)	…
1.2–1.29 mmol/L	52 716 (9.8)	52 142 (9.8)	574 (10.0)	…
0.9–1.19 mmol/L	144 059 (26.7)	142 472 (26.7)	1587 (27.6)	…
<0.9 mmol/L	45 987 (8.5)	45 422 (8.5)	565 (9.8)	…
Missing	17 746 (3.3)	17 562 (3.3)	184 (3.2)	…
Renal function (eGFR), n (%)				
≥90 mL/min	348 838 (64.7)	345 633 (64.8)	3205 (55.7)	0.215
60–89 mL/min	155 670 (28.9)	153 615 (28.8)	2055 (35.7)	…
<60 mL/min	16 131 (3.0)	15 808 (3.0)	323 (5.6)	…
Missing	18 378 (3.4)	18 211 (3.4)	167 (2.9)	…
Receiving antiplatelet drug, n (%)				
Yes	19 406 (3.6)	19 169 (3.6)	237 (4.1)	0.027
Receiving lipid-lowering drug, n (%)				
Yes	68 493 (12.7)	67 774 (12.7)	719 (12.5)	0.006
ART regimen with tenofovir alafenamide, n (%)				
Yes	86 652 (16.1)	84 844 (15.9)	1808 (31.4)	0.372
ART regimen with abacavir, n (%)				
Yes	109 609 (20.3)	107 706 (20.2)	1903 (33.1)	0.295

Abbreviations: ART, antiretroviral therapy; eGFR, estimated glomerular filtration rate; INSTI, integrase strand inhibitor; IQR, interquartile range.

During follow-up in the ITT analysis, 507 CVD events occurred, including 145 myocardial infarctions, 125 strokes, and 237 invasive cardiovascular procedures. This corresponded to 17 815 CVD events across all sequential person-trials in the non-INSTI–based ART arm, with a median time to event of 35 months (IQR: 18–53). In the INSTI-based ART arm, 222 CVD events were recorded with a median time to event of 30 months (IQR: 16–50). In the PP analysis, 462 CVD events were recorded, corresponding to 10 943 events in the non-INSTI arm over all sequential trials and 197 events in the INSTI arm.

**Figure 2.**
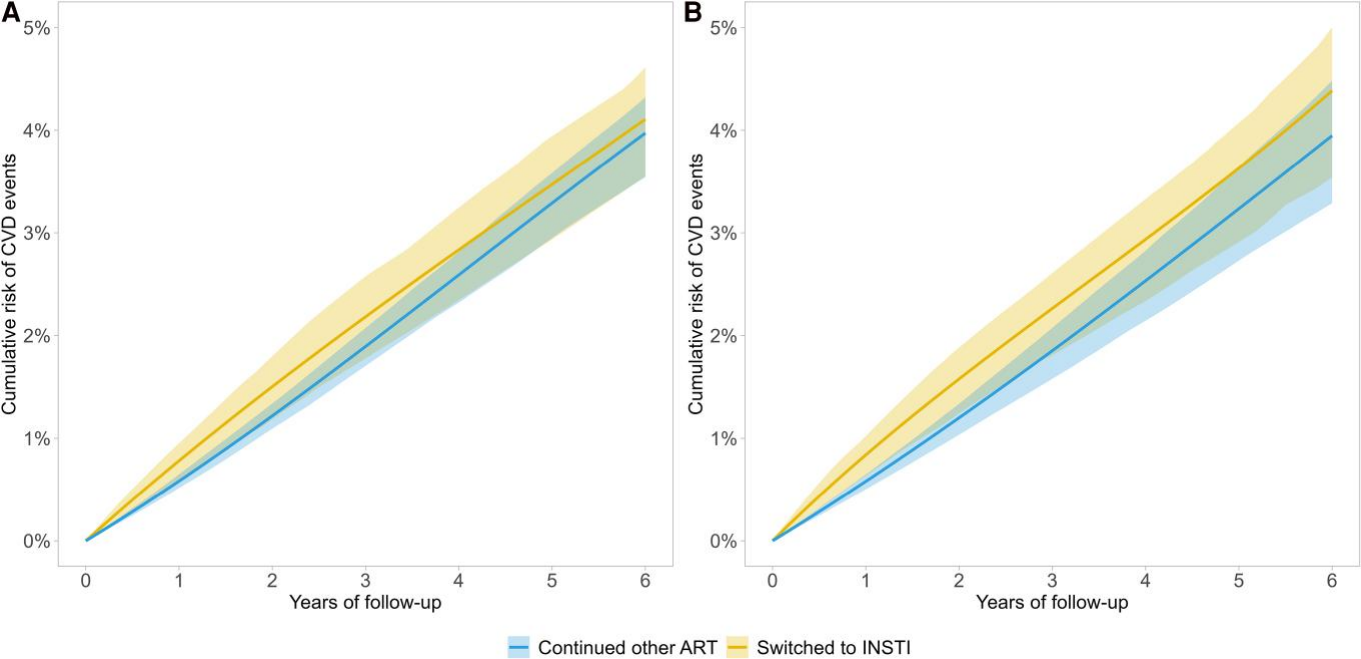
Estimated cumulative incidence of cardiovascular events in antiretroviral-experienced people with HIV showing the (*A*) intention-to-treat analysis and (*B*) per-protocol analysis. Shaded areas represent 95% bootstrap confidence intervals. Estimated adjusted cumulative cardiovascular disease (CVD) event risk curves are estimated from individuals who switched to an integrase strand transfer inhibitor (INSTI)-based regimen (N = 5750) compared with those who stayed on a non-INSTI–based regimen (N = 533 267). ART, antiretroviral therapy.

In the ITT analysis, switching to INSTI-based ART versus remaining on non-INSTI ART did not increase the 6-year cumulative CVD risk (aRR, 1.03; 95% CI, 0.88–1.16; aRD, 0.13 percentage points [pp]; 95% CI, −0.49 to 0.50; Table 2, Figure 2*A*). However, 1 year after switching, CVD risk was higher among individuals who switched (1-year aRR, 1.34; 95% CI, 1.03–1.69; aRD, 0.20 pp; 95% CI, 0.02–0.37). Results of the PP analysis were consistent with ITT findings, supporting the robustness of the observed effect to adherence (6-year aRR, 1.11; 95% CI, 0.90–1.35; 6-year aRD, 0.44 pp; 95% CI, −0.45 to 1.29, 1-year aRR, 1.44; 95% CI, 1.06–1.90; 1-year aRD, 0.26 pp; 95% CI, 0.04–0.50; Table 2, Figure 2*B*). Full model estimates are shown in Supplementary Tables 4–7.

**Table 2.** Estimated Adjusted Risks of Cardiovascular Events, Risk Ratios, and Risk Differences in Antiretroviral-experienced People With HIV

Analysis	Time (y)	Switch to INSTI-based ART (95% CI)	Remaining On Non-INSTI–based ART (95% CI)	Risk Ratio (95% CI)	Risk Difference (95% CI)
**Intention to treat**	1	0.78% (0.58–0.95)	0.58% (0.52–0.65)	1.34 (1.03–1.69)	0.20 pp (0.02–0.37)
	2	1.50% (1.19–1.80)	1.22% (1.10–1.34)	1.23 (0.99–1.49)	0.28 pp (−0.01 to 0.59)
	4	2.83% (2.35–3.24)	2.59% (2.32–2.81)	1.09 (0.93–1.25)	0.24 pp (−0.18 to 0.65)
	6	4.11% (3.55–4.61)	3.97% (3.54–4.32)	1.03 (0.88–1.16)	0.13 pp (−0.49 to 0.58)
**Per protocol**	1	0.84% (0.63–1.02)	0.58% (0.50–.65)	1.44 (1.06–1.90)	0.26 pp (0.04–0.50)
	2	1.58% (1.24–1.88)	1.20% (1.04–1.34)	1.31 (0.98–1.68)	0.38 pp (−0.03 to 0.77)
	4	2.94% (2.34–3.33)	2.53% (2.15–2.83)	1.16 (0.91–1.44)	0.40 pp (−0.23 to 1.01)
	6	4.39% (3.54–5.00)	3.95% (3.29–4.48)	1.11 (0.90–1.35)	0.44 pp (−0.45 to 1.29)

Estimates are given for each year of the 6-year follow-up for both intention-to-treat and per-protocol analyses.

Abbreviations: ART, antiretroviral treatment; CI, confidence interval; INSTI, integrase strand inhibitor; pp, percentage points.

No significant interactions were observed between the intervention and the subgroups defined by age, sex, or baseline ABC use, suggesting that the intervention's effect was consistent across these subgroups (Supplementary Table 8). Results remained unchanged when we censored individuals at raltegravir start, indicating that the findings were not driven alone by this subgroup (Supplementary Table 9, Supplementary Figure 1).

## DISCUSSION

After emulating sequential target trials using data from 8815 treatment-experienced PWH in the nationwide SHCS, we found no evidence of an increased 6-year risk of CVD among individuals who switched to an INSTI-based ART treatment compared with those who remained on non-INSTI–based ART. Both ITT and PP analyses suggested a slightly elevated CVD risk during the first year following the switch to INSTI-based ART, which decreased over subsequent follow-up.

The finding of no increased risk at 6 years is consistent with previous evidence from Europe, North America, and Australia showing no long-term difference in CVD risk in both treatment-experienced individuals [8] and ART-naive populations [7].

For early risk, our results were also consistent with risk curves previously reported [6, 8]. Of note is that the 1-year absolute risk difference remained small, at 0.2 pps. Although the corresponding risk ratio of 1.34 appears high, it should be interpreted considering the low absolute event rate, resulting in a small excess risk in absolute terms. Nevertheless, this early, transient increase in risk warrants consideration of potential underlying mechanisms and alternative explanations. Mechanisms related to atherosclerosis, including lipid increases following discontinuation of tenofovir disoproxil fumarate from non-INSTI regimens or INSTI-related weight gain, are unlikely to explain this pattern, as atherosclerotic disease typically develops slowly over many years. Average weight gain 1 year after switching to an INSTI in our study population was modest, at 1.2 kg. More acute pathways may therefore play a role. For example, initiation of INSTI-based ART could trigger early metabolic or hemodynamic changes, although these mechanisms remain speculative. Supporting this hypothesis, a recent target trial emulation conducted in West Africa found that switching to dolutegravir-based ART was associated with an increased risk of incident hypertension [18]. Alternatively, residual confounding related to unmeasured factors influencing clinicians’ decisions to switch ART may partly explain the observed early risk increase. For instance, clinicians may have switched individuals with anticipated changes in cardiometabolic profiles or prior to planned surgical intervention, potentially leading to reverse causation, where underlying risk, rather than the INSTI switch itself, contributed to early cardiovascular events. However, whether the 1-year risk difference is clinically meaningful should be interpreted in the context of its small magnitude and the well-established benefits of INSTIs when making clinical decisions.

The main strength of our study is its grounding in the SHCS, a nationally representative cohort that prospectively collects high-quality longitudinal data with standardized and comprehensive CVD outcome assessment. This minimizes misclassification bias. Use of a single national cohort allowed us to overcome inherent limitations of multinational collaborations, including heterogeneous data collection practices, heterogeneous healthcare systems, and overlapping and selective participant inclusion, which may introduce bias [6, 8]. Methodologically, emulating a sequence of target trials constitutes a second key strength, as it mitigates bias arising from the absence of a natural time zero. By following protocols used in prior target trial emulation studies [7, 8], our design also enhances comparability with existing evidence.

We also acknowledge several limitations. First, our intervention was defined pragmatically and considered switching to an INSTI class-based treatment rather than to individual INSTI drugs. Individual INSTIs have evolved over time and may have different effects on cardiovascular risk [19]. Notably, raltegravir was the first INSTI to become widely available and was initially prescribed primarily to individuals with virological failure or more advance disease. Our study, however, focused on virally suppressed individuals, and potential bias related to raltegravir use was not supported by our sensitivity analysis. Second, because ART treatments consist of drug combinations, co-administered agents may also influence CVD risk. For example, ABC is a nucleoside reverse transcriptase inhibitor, which was frequently combined with INSTIs until an increased CVD risk due to ABC became evident [17, 20, 21]. In this study, we addressed confounding by ABC through baseline adjustment. Disentangling individual drug effects would require analyses of single-drug switches, which are challenging in HIV research. Third, as with all observational studies, causal inference remains sensitive to unmeasured confounding. Lifestyle factors and aspects of patient–clinician decision-making were not captured in the available data and may therefore have contributed to residual confounding. However, the most well-known confounders were adjusted for in our analysis.

Interpreting our findings requires consideration of the broader literature on INSTI and CVD, which is complicated by the substantial participant overlap across multinational HIV cohorts, and heterogeneity in methodological approaches and healthcare systems [6–9]. These factors have hindered clear causal interpretation and may partly explain the persistent uncertainty regarding early excess risk. Further investigation, including a detailed review of clinical records beyond cohort data or laboratory-based pathogenesis studies to establish causal mechanisms, is needed to better understand the context underlying early excess risk and to confirm our findings. Based on current evidence, we do not foresee any changes to clinical guidelines at this stage.

To conclude, switching to an INSTI-based treatment in routine Swiss practice did not increase long-term CVD risk in people with well-controlled HIV. Although a slightly elevated absolute risk difference was observed early after switching, its magnitude was small. Using high-quality representative national data and target trial emulation, this analysis adds important reassuring evidence regarding prior analyses and INSTI cardiovascular safety.

## Supplementary Material

ofag479_Supplementary_Data

## Supplementary Data

Supplementary materials are available at *Open Forum Infectious Diseases* online. Consisting of data provided by the authors to benefit the reader, the posted materials are not copyedited and are the sole responsibility of the authors, so questions or comments should be addressed to the corresponding author.
