# Supplementary material for: Switching to Integrase Inhibitors and Cardiovascular Disease Risk in Treatment-Experienced People With HIV: A Sequential Target Trial Emulation in the Swiss HIV Cohort Study
Source: Open Forum Infect Dis. 2026 Aug 7;13(8):ofag479. doi: 10.1093/ofid/ofag479 (PMC13457915; doi:10.1093/ofid/ofag479)
Supplement: Supplementary file 1 [file ofag479_supplementary_data.docx]

**Supplementary appendix to “Lee at al., Switching to Integrase Inhibitors and Cardiovascular Disease Risk in Treatment-Experienced People with HIV: A Sequential Target Trial Emulation in the Swiss HIV Cohort Study”**

*Eligibility Criteria*

We assessed eligibility among all SHCS participants at the start of each calendar month, corresponding to an emulated target trial. Participants meeting eligible criteria at multiple time points could therefore contribute to multiple sequential trials. Eligibility criteria included being 18 years or older, having no history of CVD, being on a modern ART regimen, INSTI-naïve, and a suppressed viral load (≤ 50copies/mL) at the most recent measurement. We considered suppressive ART regimens having at least three ART drugs with at least two active classes or being on an INSTI or PI with at least one ART drug from another class. This study focuses on a treatment-simplification scenario, in which individuals on a stable and effective regimen were eligible to switch to the latest state-of-the-art regimen. Individuals with unsuppressed viral load were excluded as active replication is indicative of treatment failure or non-adherence.

*Treatment Strategies*

We considered two treatments strategies: switching to an INSTI-based ART or remaining on a non-INSTI-based ART. INSTI-based ART included any INSTI drug, regardless of other ART components, while a non-INSTI-based ART included all drug combinations not including an INSTI drug. The non-INSTI-based ART strategy allows participants to move between non-INSTI drug classes (eg. protease inhibitor to non-nucleoside reverse transcriptase inhibitor). As being INSTI-naïve was an inclusion criterion, an individual could contribute to multiple person-trials in the non-INSTI-based ART strategy, but maximally one person-trial in the INSTI-based ART strategy.

*Outcomes*

The outcome of interest was the occurrence of a first CVD event during follow-up, defined as a composite of myocardial infarction, stroke (ischemic or haemorrhagic), or an invasive cardiovascular procedure (carotid endarterectomy, coronary angioplasty or stenting, coronary artery by-pass grafting, or procedures on other arteries). CVD events were recorded through a dedicated case report form and verified by a senior HIV physician at the respective cohort centre. Central validation was subsequently performed using a standardized algorithm, blinded to individuals’ ART histories (DOI: 10.1161/01.cir.90.1.583).

Follow-up

For each sequential trial, eligible participants were followed from the first day of the respective trial month, corresponding to trial baseline and treatment assignment, until first CVD event, death, loss to follow-up, or administrative censoring at 6-years follow-up, whichever came first. Loss to follow-up was defined according to interrupting ART for more than two months during follow-up or cohort-specific criteria, including relocation, withdrawal, or non-response to repeated invitations.

**Table S1**. Target trial protocol

|  | **Target trial specification** | **Target trial emulation** |
| --- | --- | --- |
| **Eligibility criteria** | - Adults aged ≥18 years with HIV  - No prior history of myocardial infarction, stroke, or invasive cardiovascular procedure  - On a non-INSTI ART regimen and virally suppressed (most recent measurement <50 cp/mL)  - No prior history of INSTI use | Same. We consider participants eligible for inclusion from 11.2011 onwards, date when INSTI were first recommended as first-line ART by the European AIDS Clinical Society guidelines |
| **Treatment strategies** | 1. Switching to INSTI-based ART  2. Remaining on non-INSTI-based ART (note that switching from non-INSTI-based ART to a different ART combination without INSTI is allowed for this strategy) | Same |
| **Assignment procedures** | Patients are randomized to switching to an INSTI ART regimen or remaining on a non INSTI ART regimen on a 1:1 allocation ratio | A new trial is emulated at the beginning of each month, starting in 11.2011 until 09.2025 (database closure). We assume that each actual treatment received in observational data at baseline is as if random, given the baseline covariates age, sex, education, race, HIV transmission risk group, family history of CVD, years on ART, smoking status, BMI, CD4 count, CD4 nadir count, total cholesterol, eGRF, hypertension, diabetes, current TAF use, current ABC use, current anti-platelet use, current lipid-lowering drug use, and baseline calendar month, and are independent of potential cardiovascular outcomes |
| **Follow-up** | Follow up begins at randomisation and ends when the participant experiences the outcome, death, loss to follow up, or at administrative censoring, whichever comes first. In the per protocol analysis, follow up additionally ends when participant deviates from their assigned treatment strategy. | Follow up for each trial begins at treatment assignment and ends when the participant experiences the outcome, death, loss to follow up, or at administrative censoring, whichever comes first. In the per protocol analysis, follow up additionally ends when participant deviates from their assigned treatment strategy. |
| **Outcomes** | Time to first cardiovascular event [composite of fatal and non-fatal myocardial infarction, stroke (cerebral infarction/haemorrhage), or invasive cardiovascular procedure (carotid endarterectomy, coronary angioplasty/stenting, coronary artery by-pass grafting, or procedures on other arteries)]. | Same |
| **Causal contrasts** | - Intention to treat effect (average effect of assigning a strategy)  - Per protocol effect (average effect of applying a strategy) | Observational analogues of both  Causal effects are averaged across sequential trials |
| **Identifying assumptions** | - Positivity: every participant has a non-null probability of being assigned to each treatment arm  -Exchangeability ensured by randomization  -Consistency: treatment strategies are well-defined  -Interference: we do not expect spillover  -No diagnostic error | - Positivity: Individuals have a non-zero probability of receiving each of the treatment strategies (inclusion in trials assessed at the start of each trial, weights are truncated)  - Exchangeability (conditional): Treatment assignment and censoring are independent of potential outcomes, conditional on the measured baseline covariates: age, sex, education, race, HIV transmission risk group, family history of CVD, years on ART, smoking status, BMI, CD4 count, CD4 nadir count, total cholesterol, eGRF, hypertension, diabetes, current TAF use, current ABC use, current anti-platelet use, current lipid-lowering drug use, and baseline calendar month  - Consistency: Treatment strategies are well defined  - Interference: Counterfactual event time for an individual does not depend on treatment received by other individuals  - Model specification: Models for the outcome, treatment weights, and censoring weights are assumed to be correctly specified. |
| **Data analysis plan** | - Use Cox proportional hazards model, and reports hazard ratio and cumulative incidence with 95% confidence intervals for the ITT and per-protocol effect | Pool data from all sequential trials and use pooled logistic regression with individual bootstrap procedures to derive risk ratio and cumulative incidence with 95% confidence intervals for the ITT and per-protocol effect.  - Intention to treat analysis: Same with monthly sequential trial emulation from 11.2011 until database closure  - Per protocol analysis: application of censoring weights to account for artificial censoring due to non-adherence, as well as loss-to follow-up. Weights are truncated.to avoid extreme value, destabilize estimator and inflate precision. |

**Table S2**. Baseline antiretroviral class, by treatment arm assignment in observed data.

| Antiretroviral treatment | Switch to INSTI-based ART n(%) | Remaining on non-INSTI-based ART n(%) |
| --- | --- | --- |
| Integrase Strand Transfer Inhibitor (INSTI)--based | 5750 (100.0%) | - |
| Protease Inhibitor (PI)-based | - | 188260 (35.3%) |
| Non-Nucleoside Reverse Transcriptase Inhibitor (NNRTI)-based | - | 340698 (63.9%) |
| Other (Fusion Inhibitor or non-INSTI trial treatment) | - | 4309 (0.8%) |

ART: antiretroviral treatment

**Table S3**. Overview of the five most common baseline antiretroviral combinations, by treatment arm assignment in observed data.

|  | Rank | Treatment regimens* | n (%) |
| --- | --- | --- | --- |
| Non-INSTI | 1 | EFV ETC TDF | 125987 (19.0%) |
|  | 2 | ETC RPV TAF | 60812 (9.2%) |
|  | 3 | ETC RPV TDF | 56981 (8.6%) |
|  | 4 | DRV ETC RTV TDF | 55915 (8.4%) |
|  | 5 | ATV ETC RTV TDF | 34745 (5.2%) |
| INSTI | 1 | 3TC ABC DTG | 110633 (24.1%) |
|  | 2 | BIC ETC TAF | 87461 (19.1%) |
|  | 3 | COB ETC EVG TAF | 60992 (13.3%) |
|  | 4 | 3TC DTG | 47517 (10.4%) |
|  | 5 | DTG ETC TAF | 38865 (8.5%) |

*3TC = lamivudine; ABC = abacavir; ATV = atazanavir; BIC = bictegravir; COB = cobicistat; DRV = darunavir; DTG = dolutegravir; EFV = efavirenz; ETC = emtricitabine; EVG = elvitegravir; RPV = rilpivirine; RTV = ritonavir; TAF = tenofovir alafenamide; TDF = tenofovir disoproxil fumarate

**Table S4**. Model parameter estimates for cardiovascular disease events in the intention-to-treat analysis.

|  | Coefficient | Lower 95% CI | Upper 95% CI |
| --- | --- | --- | --- |
| Intercept | -9.429 | -9.622 | -9.236 |
| Treatment arm: INSTI | 0.054 | -0.079 | 0.188 |
| Follow-up time (quadratic): linear component | 543.624 | 457.370 | 629.877 |
| Follow-up time (quadratic): quadratic component | -204.580 | -289.020 | -120.141 |
| Calendar month (quadratic): linear component | -1053.962 | -1171.186 | -936.738 |
| Calendar month (quadratic): quadratic component | 59.610 | -34.114 | 153.334 |
| Sex: Female | -0.611 | -0.657 | -0.564 |
| Race: African ancestry | -0.229 | -0.304 | -0.154 |
| Race: Other | -0.882 | -0.991 | -0.772 |
| Race: Unknown | 0.574 | 0.262 | 0.885 |
| HIV transmission group: Men having sex with men | -0.023 | -0.061 | 0.015 |
| HIV transmission group: Person who injects drugs | 0.174 | 0.125 | 0.222 |
| HIV transmission group: Other | -0.385 | -0.480 | -0.290 |
| Age (years) | 0.061 | 0.059 | 0.063 |
| CD4 count: 350-499 cells/uL | -0.200 | -0.242 | -0.157 |
| CD4 count: 200-349 cells/uL | 0.115 | 0.065 | 0.165 |
| CD4 count: <200 cells/uL | 0.060 | -0.038 | 0.157 |
| CD4 count: Missing | 0.130 | -0.309 | 0.570 |
| CD4 count nadir category: 350-499 cells/uL | 0.940 | 0.843 | 1.037 |
| CD4 count nadir category: 200-349 cells/uL | 0.622 | 0.531 | 0.713 |
| CD4 count nadir category: <200 cells/uL | 0.511 | 0.419 | 0.602 |
| CD4 count nadir category: Missing | -0.920 | -1.608 | -0.233 |
| BMI: Normal (18.5-24.9 kg/m2) | -0.062 | -0.137 | 0.014 |
| BMI: Overweight (25-29.9 kg/m2) | -0.118 | -0.197 | -0.038 |
| BMI: Obese (≥30 kg/m2) | -0.234 | -0.323 | -0.145 |
| BMI: Unknown | -0.682 | -1.264 | -0.100 |
| Hypertension: No | -0.326 | -0.357 | -0.294 |
| Cholesterol: 4.1-5.19 | -0.189 | -0.238 | -0.140 |
| Cholesterol: 5.2-6.19 | -0.016 | -0.066 | 0.033 |
| Cholesterol: 6.2-7.2 | 0.300 | 0.244 | 0.355 |
| Cholesterol: >7.2 | 0.370 | 0.291 | 0.449 |
| Cholesterol: Missing | -0.211 | -0.500 | 0.078 |
| Diabetes: No | -0.228 | -0.282 | -0.173 |
| Renal function (eGRF): 60-89 mL/min | 0.128 | 0.094 | 0.163 |
| Renal function (eGRF): <60 mL/min | 0.097 | 0.033 | 0.160 |
| Renal function (eGRF): Missing | 0.466 | 0.266 | 0.667 |
| Currently smoking: No | -0.757 | -0.791 | -0.723 |
| Currently smoking: Missing | 0.737 | 0.158 | 1.316 |
| ART regimen with TAF: No | 0.092 | 0.025 | 0.160 |
| ART regimen with ABC: No | -0.576 | -0.608 | -0.543 |
| Highest education achieved: Basic education | 0.361 | 0.326 | 0.397 |
| Highest education achieved: No professional education | 0.235 | 0.158 | 0.313 |
| Highest education achieved: Unknown | -0.628 | -0.750 | -0.505 |
| Family history of CVD: No | -0.329 | -0.371 | -0.288 |
| Family history of CVD: Unknown | -0.663 | -0.792 | -0.533 |
| Receiving anti-platelet drug: No | -0.599 | -0.646 | -0.551 |
| Receiving lipid-lowering drug: No | -0.199 | -0.236 | -0.162 |
| Years on ART | 0.030 | 0.027 | 0.033 |
| Interaction: INSTI arm * Calendar month (quadratic): linear component | -751.708 | -1502.381 | -1.035 |
| Interaction: INSTI arm * Calendar month (quadratic): quadratic component | 339.747 | -412.519 | 1092.013 |

**Table S5**. Model parameter estimates for censoring weights due to treatment non-adherence in the per-protocol analysis.

|  | Coefficient | Lower 95% CI | Upper 95% CI |
| --- | --- | --- | --- |
| Intercept | -2.637 | -2.965 | -2.310 |
| Calendar month (quadratic): linear component | 1.604 | -31.653 | 34.862 |
| Calendar month (quadratic): quadratic component | -429.161 | -454.830 | -403.491 |
| Sex: Female | 0.149 | 0.072 | 0.225 |
| Race: African ancestry | 0.037 | -0.053 | 0.128 |
| Race: Other | 0.048 | -0.050 | 0.147 |
| Race: Unknown | -0.540 | -1.349 | 0.269 |
| HIV transmission group: Men having sex with men | 0.145 | 0.070 | 0.220 |
| HIV transmission group: Person who injects drugs | 0.008 | -0.092 | 0.107 |
| HIV transmission group: Other | 0.158 | 0.033 | 0.283 |
| Age (years) | -0.005 | -0.008 | -0.002 |
| Years on ART | 0.004 | -0.001 | 0.009 |
| BMI: Normal (18.5-24.9 kg/m2) | 0.056 | -0.087 | 0.199 |
| BMI: Overweight (25-29.9 kg/m2) | -0.004 | -0.153 | 0.144 |
| BMI: Obese (≥30 kg/m2) | 0.025 | -0.136 | 0.187 |
| BMI: Unknown | 0.443 | -0.498 | 1.385 |
| CD4 count: 350-499 cells/uL | 0.031 | -0.042 | 0.103 |
| CD4 count: 200-349 cells/uL | 0.061 | -0.042 | 0.163 |
| CD4 count: <200 cells/uL | 0.540 | 0.375 | 0.704 |
| CD4 count: Missing | 0.775 | 0.054 | 1.497 |
| CD4 count nadir category: 350-499 cells/uL | -0.068 | -0.191 | 0.055 |
| CD4 count nadir category: 200-349 cells/uL | -0.050 | -0.159 | 0.060 |
| CD4 count nadir category: <200 cells/uL | -0.021 | -0.135 | 0.092 |
| CD4 count nadir category: Missing | -0.547 | -1.424 | 0.330 |
| Hypertension: No | -0.004 | -0.065 | 0.057 |
| Cholesterol: 4.1-5.19 | -0.085 | -0.166 | -0.004 |
| Cholesterol: 5.2-6.19 | -0.084 | -0.169 | 0.002 |
| Cholesterol: 6.2-7.2 | -0.156 | -0.261 | -0.051 |
| Cholesterol: >7.2 | -0.095 | -0.252 | 0.061 |
| Cholesterol: Missing | 0.854 | 0.342 | 1.365 |
| Diabetes: No | -0.092 | -0.213 | 0.029 |
| Renal function (eGRF): 60-89 mL/min | 0.357 | 0.294 | 0.420 |
| Renal function (eGRF): <60 mL/min | 0.675 | 0.550 | 0.800 |
| Renal function (eGRF): Missing | -0.781 | -1.246 | -0.317 |
| Currently smoking: No | -0.128 | -0.189 | -0.067 |
| Currently smoking: Missing | -0.421 | -1.419 | 0.576 |
| ART regimen with TAF: No | -1.420 | -1.505 | -1.335 |
| ART regimen with ABC: No | -1.101 | -1.166 | -1.036 |
| Highest education achieved: Basic education | 0.050 | -0.011 | 0.111 |
| Highest education achieved: No professional education | -0.094 | -0.214 | 0.025 |
| Highest education achieved: Unknown | 0.120 | -0.054 | 0.294 |
| Family history of CVD: No | -0.145 | -0.230 | -0.060 |
| Family history of CVD: Unknown | -0.103 | -0.268 | 0.062 |
| Receiving anti-platelet drug: No | 0.007 | -0.132 | 0.147 |
| Receiving lipid-lowering drug: No | 0.133 | 0.045 | 0.220 |

**Table S6**. Model parameter estimates for censoring weights for loss to follow-up in the per-protocol analysis.

|  | Coefficient | Lower 95% CI | Upper 95% CI |
| --- | --- | --- | --- |
| Intercept | 5.767 | 5.493 | 6.041 |
| Calendar month (quadratic): linear component | -778.855 | -811.087 | -746.624 |
| Calendar month (quadratic): quadratic component | -1322.660 | -1352.505 | -1292.816 |
| Sex: Female | 0.014 | -0.052 | 0.079 |
| Race: African ancestry | -0.036 | -0.111 | 0.040 |
| Race: Other | -0.030 | -0.112 | 0.052 |
| Race: Unknown | 0.004 | -0.644 | 0.651 |
| HIV transmission group: Men having sex with men | 0.032 | -0.033 | 0.096 |
| HIV transmission group: Person who injects drugs | -0.193 | -0.277 | -0.110 |
| HIV transmission group: Other | 0.069 | -0.036 | 0.175 |
| Age (years) | -0.002 | -0.005 | 0.001 |
| Years on ART | 0.007 | 0.002 | 0.011 |
| BMI: Normal (18.5-24.9 kg/m2) | 0.239 | 0.113 | 0.364 |
| BMI: Overweight (25-29.9 kg/m2) | 0.229 | 0.100 | 0.359 |
| BMI: Obese (≥30 kg/m2) | 0.261 | 0.124 | 0.399 |
| BMI: Unknown | -0.645 | -1.379 | 0.089 |
| CD4 count: 350-499 cells/uL | -0.050 | -0.117 | 0.016 |
| CD4 count: 200-349 cells/uL | -0.269 | -0.359 | -0.178 |
| CD4 count: <200 cells/uL | -0.800 | -0.941 | -0.660 |
| CD4 count: Missing | -1.213 | -3.336 | 0.909 |
| CD4 count nadir category: 350-499 cells/uL | 0.067 | -0.036 | 0.170 |
| CD4 count nadir category: 200-349 cells/uL | 0.080 | -0.012 | 0.173 |
| CD4 count nadir category: <200 cells/uL | 0.097 | 0.002 | 0.193 |
| CD4 count nadir category: Missing | 8.850 | -100.954 | 118.653 |
| Hypertension: No | -0.053 | -0.102 | -0.005 |
| Cholesterol: 4.1-5.19 | 0.156 | 0.094 | 0.217 |
| Cholesterol: 5.2-6.19 | 0.270 | 0.202 | 0.338 |
| Cholesterol: 6.2-7.2 | 0.276 | 0.187 | 0.364 |
| Cholesterol: >7.2 | 0.275 | 0.125 | 0.425 |
| Cholesterol: Missing | 0.264 | -0.946 | 1.475 |
| Diabetes: No | -0.014 | -0.089 | 0.061 |
| Renal function (eGRF): 60-89 mL/min | -0.021 | -0.075 | 0.033 |
| Renal function (eGRF): <60 mL/min | -0.049 | -0.143 | 0.046 |
| Renal function (eGRF): Missing | 0.830 | -0.081 | 1.740 |
| Currently smoking: No | 0.130 | 0.078 | 0.183 |
| Currently smoking: Missing | 3.563 | 1.959 | 5.168 |
| ART regimen with TAF: No | -0.084 | -0.134 | -0.033 |
| ART regimen with ABC: No | 0.133 | 0.055 | 0.211 |
| Highest education achieved: Basic education | 0.009 | -0.042 | 0.060 |
| Highest education achieved: No professional education | -0.008 | -0.106 | 0.091 |
| Highest education achieved: Unknown | -0.131 | -0.276 | 0.014 |
| Family history of CVD: No | 0.013 | -0.061 | 0.088 |
| Family history of CVD: Unknown | -0.043 | -0.183 | 0.097 |
| Receiving anti-platelet drug: No | 0.010 | -0.094 | 0.113 |
| Receiving lipid-lowering drug: No | -0.043 | -0.103 | 0.017 |
| INSTI treatment | 0.038 | -0.015 | 0.091 |

**Table S7**. Model parameter estimates for cardiovascular disease events in the per-protocol analysis

|  | Coefficient | Lower 95% CI | Upper 95% CI |
| --- | --- | --- | --- |
| Intercept | -7.514 | -7.529 | -7.499 |
| Treatment arm: INSTI | 0.153 | 0.128 | 0.178 |
| Follow-up time (quadratic): linear component | 396.710 | 330.064 | 463.357 |
| Follow-up time (quadratic): quadratic component | -68.258 | -129.739 | -6.778 |
| Calendar month (quadratic): linear component | -356.819 | -413.670 | -299.968 |
| Calendar month (quadratic): quadratic component | -36.156 | -92.634 | 20.323 |
| Interaction: INSTI arm * Calendar month (quadratic): linear component | -615.261 | -723.938 | -506.584 |
| Interaction: INSTI arm * Calendar month (quadratic): quadratic component | 387.093 | 281.215 | 492.971 |

CI: confidence interval, INSTI: integrase strand inhibitor

**Table S8**. Subgroups intention-to-treat effect of switching to an integrase strand inhibitor in subgroups on cardiovascular disease risk.

| Subgroup | Odds ratio for cardiovascular disease | Lower 95% CI | Upper 95% CI | Interaction p-value* |
| --- | --- | --- | --- | --- |
| **Age (continuous)** |  |  |  | 0.163 |
| Below 65 years | 1.065 | 0.912 | 1.243 | - |
| 65 years old or above | 0.886 | 0.643 | 1.221 | - |
| **Sex** |  |  |  | 0.203 |
| Female | 0.817 | 0.563 | 1.185 | - |
| Male | 1.102 | 0.955 | 1.272 | - |
| **Abacavir use** |  |  |  | 0.271 |
| Start regimen without abacavir | 1.111 | 0.928 | 1.330 | - |
| Start regimen with abacavir | 0.890 | 0.727 | 1.090 | - |

*P-values derived from treatment*subgroup interaction term in the pooled ITT model.

Non-significant interaction terms and wide confidence intervals of subgroups suggest no evidence of a subgroup effect.

**Table S9**. Estimated adjusted risks of cardiovascular events, risk ratios and risk differences in antiretroviral-experienced people living with HIV for the intention-to-treat analysis excluding individual initiating raltegravir and censoring at time of raltegravir switch.

| **Analysis** | **Time (years)** | **Switch to INSTI-based ART (95% CI)** | **Remaining on non-INSTI-based ART (95% CI)** | **Risk Ratio (95% CI)** | **Risk Difference (95% CI)** |
| --- | --- | --- | --- | --- | --- |
| Intention to treat | 1 | 0.78% (0.56 - 0.96) | 0.57% (0.50 - 0.65) | 1.36 (0.97 - 1.67) | 0.21% pp (-0.02 - 0.38) |
|  | 2 | 1.53% (1.19 - 1.81) | 1.20% (1.08 - 1.35) | 1.27 (0.99 - 1.51) | 0.32% pp (-0.01 - 0.59) |
|  | 4 | 2.93% (2.38 - 3.38) | 2.58% (2.31 - 2.91) | 1.13 (0.96 - 1.29) | 0.35% pp (-0.11 - 0.74) |
|  | 6 | 4.19% (3.50 - 4.73) | 3.99% (3.61 - 4.44) | 1.05 (0.91 - 1.18) | 0.20% pp (-0.37 - 0.71) |

ART: antiretroviral treatment; CI: confidence interval; pp: percentage points

**Figure S1**. Sensitivity analysis censoring individuals at raltegravir initiation: Estimated adjusted cumulative cardiovascular event risk curves for individuals who switched to an INSTI-based regimen (N = 5,067) compared to those who stayed on a non-INSTI-based regimen (N = 533,059). 95% confidence intervals were derived from 200 bootstrap samples.
